# Fetal age assessment based on 2^nd ^trimester ultrasound in Africa and the effect of ethnicity

**DOI:** 10.1186/1471-2393-8-48

**Published:** 2008-10-30

**Authors:** Daniel Salpou, Torvid Kiserud, Svein Rasmussen, Synnøve Lian Johnsen

**Affiliations:** 1 Centre for International Health, University of Bergen, Norway; 2 Ngoundere Protestant Hospital, Ngoundere, Cameroon; 3 Department of Obstetrics and Gynecology, Haukeland University Hospital, Bergen, Norway; 4 Department of Clinical Medicine, University of Bergen, Norway; 5 Medical Birth Registry of Norway, Locus of Registry Based Epidemiology, University of Bergen and the Norwegian Institute of Public Health, Norway

## Abstract

**Background:**

The African population is composed of a variety of ethnic groups, which differ considerably from each other. Some studies suggest that ethnic variation may influence dating. The aim of the present study was to establish reference values for fetal age assessment in Cameroon using two different ethnic groups (Fulani and Kirdi).

**Methods:**

This was a prospective cross sectional study of 200 healthy pregnant women from Cameroon. The participants had regular menstrual periods and singleton uncomplicated pregnancies, and were recruited after informed consent. The head circumference (HC), outer-outer biparietal diameter (BPDoo), outer-inner biparietal diameter and femur length (FL), also called femur diaphysis length, were measured using ultrasound at 12–22 weeks of gestation. Differences in demographic factors and fetal biometry between ethnic groups were assessed by t- and Chi-square tests.

**Results:**

Compared with Fulani women (N = 96), the Kirdi (N = 104) were 2 years older (p = 0.005), 3 cm taller (p = 0.001), 6 kg heavier (p < 0.0001), had a higher body mass index (BMI) (p = 0.001), but were not different with regard to parity. Ethnicity had no effect on BPDoo (p = 0.82), HC (p = 0.89) or FL (p = 00.24). Weight, height, maternal age and BMI had no effect on HC, BPDoo and FL (p = 0.2–0.58, 0.1–0.83, and 0.17–0.6, respectively).

When comparing with relevant European charts based on similar design and statistics, we found overlapping 95% CI for BPD (Norway & UK) and a 0–4 day difference for FL and HC.

**Conclusion:**

Significant ethnic differences between mothers were not reflected in fetal biometry at second trimester. The results support the recommendation that ultrasound in practical health care can be used to assess gestational age in various populations with little risk of error due to ethnic variation.

## Background

Maternal and perinatal mortalities in sub-Saharan countries are among the highest in the world. In Cameroon the maternal mortality rate is 430 per 100 000 live births and the infant mortality rate 87 per 1000 under one year) [1]. Gestational age (GA) has emerged as one of the most important predictors of perinatal mortality and morbidity [2]. By combining GA and fundal height of the uterus, complications such as intra-uterine growth restriction (IUGR), oligohydramnion, macrosomia, multiple pregnancy and polyhydramnion may be identified [3]. Knowledge of GA is also a prerequisite in the management of conditions such as premature rupture of membranes, preterm labour, post dates, antepartum bleeding, preeclampsia etc.

Last menstrual period (LMP) is simple and the most common method of calculating GA. However, 45–68% of pregnant women have been reported to have irregular periods or uncertain information regarding their LMP. The ultrasonographic measurements of the fetal BPD is a more reliable method, predicting date of spontaneous delivery with greater certainty than even a certain LMP [4-7]. Ultrasound is particularly useful in parts of the world where women often cannot account for their LMP [8,9]. In Cameroon the illiteracy rate is 68% among people older than 15 years [1], and many of the pregnant women who attend hospital clinics do not know the exact date of their LMP, but they count completed months.

Biparietal diameter (BPD) is the most commonly used ultrasound measurement for fetal age assessment. Fetal age assessment in the second trimester can also be based on head circumference (HC) and femur length (FL). These methods are less influenced by maternal and fetal factors such as parity, age and fetal gender [10-15].

The African population is composed of a variety of ethnic groups, differing considerably from each other. Some studies suggest that population differences in fetal biometry are negligible and that separate studies are not essential [16,17]. Other studies however demonstrate morphometric variation among different population groups around the world [18-20] suggesting that ethnic variation may influence dating.

The aim of the present study was therefore to establish reference values for fetal age assessment in Cameroon using two different ethnic groups. We also wanted to determine the effect of maternal morphometry on the age assessment and to compare these new reference charts with other relevant charts.

## Methods

This was a prospective cross sectional study of 200 pregnant women belonging to two different ethnic groups in the northern part of Cameroon: the Fulani people who are slender, and the Kirdi people who are in general stocky (Table 1). The participants were recruited from an antenatal clinic when they attended their routine prenatal care at 12–22 weeks of gestation. About 20 participants were recruited for each gestational week. The women were healthy with regular menstrual periods and certain information about LMP. They participated voluntarily after informed consent according to a protocol acknowledged by the hospital committee of Medical Research Ethics.

**Table 1:** Comparison between Fulani (N = 96) and Kirdi (N = 104) according to height, weight, body mass index (BMI), maternal age (A = ANOVA test).

Height	Mean	95% Confidence Interval		*p *= 0.001
Fulani	1.57	1.56	1.58	

Kirdi	1.595	1.585	1.605	

Total	1.58	1.57	1.59	

Weight	Mean	95% Confidence Interval		*p *< 0.0001

Fulani	59.93	57.90	61.95	

Kirdi	65.87	63.93	67.82	

Total	62.90	61.50	64.30	

BMI	Mean	95% Confidence Interval		*p *= 0.001

Fulani	24.03	23.26	24.80	

Kirdi	25.83	25.09	26.57	

Total	24.93	24.40	25.46	

Age	Mean	95% Confidence Interval		*p *= 0.005

Fulani	25.32	24.19	26.45	

Kirdi	27.59	26.50	28.67	

Total	26.46	26.67	27.24	

One investigator (DS) trained at a Norwegian university hospital did all the ultrasound examinations using Shimasonic (Shimadzu) SDL-300, Japan, with a 3.5 MHz curvilinear probe.

Fetal head measurements were obtained in a horizintal section at the level of the thalamus and the cavum septi pellucidi [21]. Measurements of the biparietal diameter were obtained by placing the callipers at the outer border of the cranium on both sides (BPD outer-outer, BPDoo) and at the leading edges (BPD outer-inner, BPDoi). The occipital-frontal diameter (OFD) was measured between the leading edge of the frontal bone and the outer border of the occiput. Head circumference (HC) was estimated from the measurement of the OFD and the BPDoo using the formula *π*(BPD+OFD)/2 [21]. The fetal femur length (FL) was obtained in a longitudinal section by placing the calliper at the end of the diaphysis on both sides [22], also called the femur diaphysis length (FDL) [23]. For each parameter three measurements were used to calculate a mean.

Gestational age was calculated from the first day of the last menstrual period, and corrected for cycle length; i.e. corresponding number of days were added or subtracted according to menstrual cycle length shorter or longer than 28 days, respectively.

### Statistics

The sample size was determined based on the power calculation and design of a corresponding previous study [24]. Fractional polynomial regression models were fitted to the data in order to construct the mean [24]. To construct the 2.5th, 5^th^, 10^th^, 25^th^, 75^th^, 90^th^, 95^th^, and 97.5^th ^centiles, the method of scaled absolute residuals was applied [25]. Differences in demographic factors and fetal biometry between ethnic groups were assessed by t- and Chi-square tests. Continuous dependent variables were power transformed to normality were necessary. Intra-observer coefficient of variation was calculated based on the three repeat measurements of each parameter in all participants. The intra-observer variation was also analyzed as the intra-class correlation. The SPSS statistical package (SPSS, Chicago, IL, USA) was used, except for the intra-observer coefficient of variation, which was carried out according to the 'logarithmic method' of Bland [26].

## Results

Table 1 and Table 2 show the characteristics of the total population and the comparison between the 96 Fulani and 104 Kirdi with regard to maternal age, height, weight, BMI and parity. Compared with the Fulani women, the Kirdi were two years older (p = 0.005), three cm taller (p = 0.001), six kg heavier (p < 0.0001), had a higher BMI (p = 0.001), but were not different with regard to parity. Seventy (35%) of all the women had never been to school and 162 (81%) of them were housewives, while only one (0.5%) of their husbands was unemployed. All the women were married.

**Table 2:** Comparison between Fulani (N = 96) and Kirdi (N = 104) according to parity (B= Chi-square test)

Parity	Fulani		Kirdi		Total		
	n	%	N	%	n	%	
0	22	22.92	24	23.08	46	24.21	Chi-square test:
1	24	25.00	26	25.00	50	26.32	*P *= 0.9
2	14	14.58	15	14.42	29	15.26	
3	13	13.54	10	9.62	23	12.11	
4	9	9.38	9	8.65	18	9.47	
5	14	14.58	20	19.23	34	17.90	

**Figure 1:**
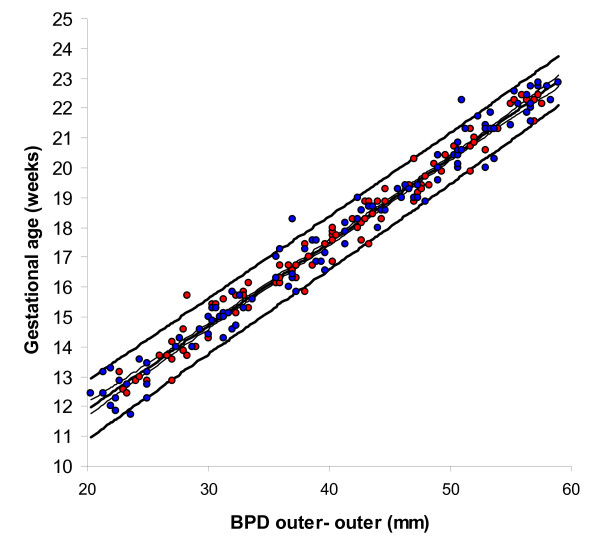
**Raw data with fitted centiles of gestational age (2.5^th^, 50^th ^and 97.5^th^) by biparietal diameter (outer-outer) and 95% CI for the mean.** Red dots are Fulani and blue dots Kirdi.

**Figure 2:**
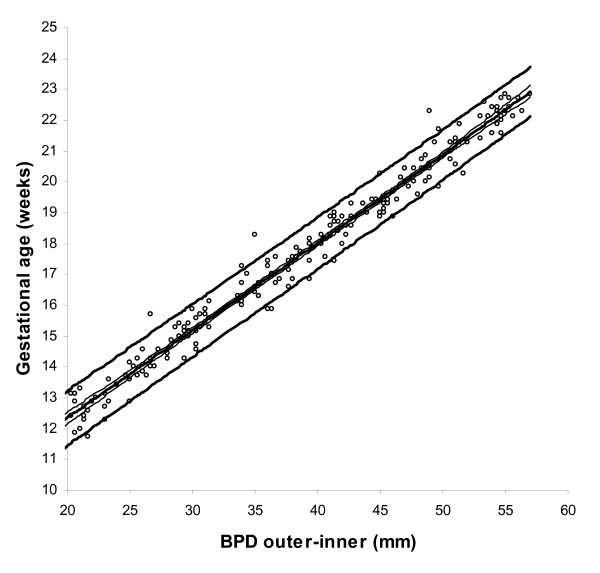
Raw data with fitted centiles of gestational age (2.5^th^, 50^th ^and 97.5^th^) by biparietal diameter (outer-inner)) and 95% CI for the mean.

**Figure 3:**
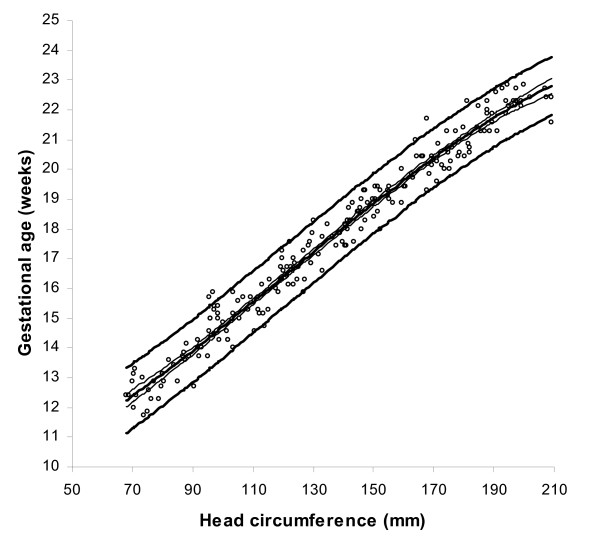
Raw data with fitted centiles of gestational age (2.5^th^, 50^th ^and 97.5^th^) by head circumference and 95% CI for the mean.

**Figure 4:**
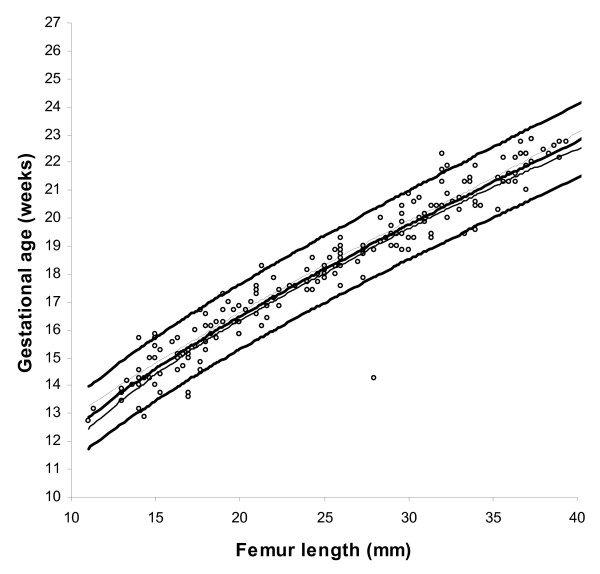
Raw data with fitted centiles of gestational age (2.5^th^, 50^th ^and 97.5%) by femur length and 95% CI for the mean.

BPD, OFD and HC were successfully determined in all participants, while in 18 cases visualisation of the FL was not possible during early pregnancy. For the ethnic groups combined, raw data with fitted 2.5^th^, 50^th ^and 97.5^th ^centiles and 95% CI for mean gestational age as functions of BPDoo, BPDoi, HC and FL are presented in Figures 1, 2, 3, 4 and the corresponding charts for GA assessment according to biometrical measurements in Tables 3, 4, 5 and 6. Mean gestational age and standard deviation (SD) as functions of the anatomical parameters were: Mean gestational age (weeks)^1.255 ^= 9.2309763061 + 0.6491253792 BPDoo (mm) + 0.0000166409 BPDoo^3^, SD = 1.1891063079 - 0.0000676577 BPDoo; mean gestational age (weeks)^1.255 ^= 9.9633718109 + 0.6655891074 BPDoi (mm) + 0.0000165695 BPDoi^3^, SD = 1.0450011947 + 0.0018760334 BPDoi; mean gestational age (weeks)^1.255 ^= 16.8857174784 + 0.0016547829 HC (mm)^2 ^- 0.0000042242 HC^3^, SD = 1.2876967006 + 0.0004840074 HC and mean gestational age (weeks)^1.255 ^= 1.2653359275 + 6.9440971709 FL (mm)^0.5 ^+ 0.0033225118 FL^2^, SD = 1.1872945587 + 0.0170042678 FL.

**Table 3:** Gestational age assessment by biparietal diameter outer-outer (BPDoo)

	Centiles					
	
	50th		2.5th		97.5th	
**BPDoo (mm)**	weeks	days	weeks	days	weeks	days

20	**11**	**6**	10	6	12	6
21	**12**	**1**	11	1	13	0
22	**12**	**3**	11	3	13	2
23	**12**	**5**	11	5	13	4
24	**13**	**0**	12	0	13	6
25	**13**	**2**	12	2	14	1
26	**13**	**4**	12	4	14	3
27	**13**	**5**	12	6	14	5
28	**14**	**0**	13	1	15	0
29	**14**	**2**	13	3	15	2
30	**14**	**4**	13	5	15	4
31	**14**	**6**	14	0	15	6
32	**15**	**1**	14	2	16	1
33	**15**	**3**	14	4	16	3
34	**15**	**5**	14	6	16	5
35	**16**	**0**	15	1	16	6
36	**16**	**2**	15	3	17	1
37	**16**	**4**	15	5	17	3
38	**16**	**6**	16	0	17	5
39	**17**	**1**	16	2	18	0
40	**17**	**3**	16	4	18	2
41	**17**	**5**	16	6	18	4
42	**18**	**0**	17	1	18	6
43	**18**	**2**	17	3	19	1
44	**18**	**4**	17	5	19	3
45	**18**	**6**	18	0	19	5
46	**19**	**1**	18	2	20	0
47	**19**	**3**	18	4	20	2
48	**19**	**5**	18	6	20	4
49	**20**	**0**	19	1	20	6
50	**20**	**2**	19	3	21	1
51	**20**	**4**	19	5	21	3
52	**20**	**6**	20	0	21	5
53	**21**	**1**	20	2	22	0
54	**21**	**3**	20	4	22	2
55	**21**	**5**	20	6	22	4
56	**22**	**0**	21	1	22	6
57	**22**	**2**	21	3	23	1
58	**22**	**4**	21	5	23	3

**Table 4:** Gestational age asssessment by biparietal diameter outer-inner (BPDoi)

	Centiles					
	
	50th		2.5th		97.5th	
**BPDoi (mm)**	weeks	days	weeks	days	weeks	days

20	**12**	**2**	11	3	13	1
21	**12**	**4**	11	5	13	3
22	**12**	**6**	12	0	13	5
23	**13**	**1**	12	2	14	0
24	**13**	**3**	12	4	14	2
25	**13**	**5**	12	6	14	4
26	**14**	**0**	13	1	14	6
27	**14**	**2**	13	3	15	1
28	**14**	**4**	13	5	15	3
29	**14**	**6**	14	0	15	5
30	**15**	**1**	14	2	16	0
31	**15**	**3**	14	4	16	2
32	**15**	**5**	14	6	16	4
33	**16**	**0**	15	1	16	6
34	**16**	**2**	15	3	17	1
35	**16**	**4**	15	5	17	3
36	**16**	**6**	16	0	17	5
37	**17**	**1**	16	2	17	6
38	**17**	**3**	16	4	18	1
39	**17**	**5**	16	6	18	3
40	**18**	**0**	17	1	18	5
41	**18**	**2**	17	3	19	0
42	**18**	**4**	17	5	19	2
43	**18**	**6**	18	0	19	4
44	**19**	**1**	18	2	19	6
45	**19**	**3**	18	4	20	1
46	**19**	**5**	18	6	20	3
47	**20**	**0**	19	1	20	5
48	**20**	**2**	19	3	21	0
49	**20**	**4**	19	5	21	2
50	**20**	**6**	20	0	21	4
51	**21**	**1**	20	2	21	6
52	**21**	**3**	20	4	22	1
53	**21**	**5**	20	6	22	3
54	**22**	**0**	21	1	22	5
55	**22**	**2**	21	3	23	1
56	**22**	**4**	21	5	23	3
57	**22**	**6**	22	0	23	5
58	**23**	**1**	22	2	24	0

**Table 5:** Gestational age ssessment by head circumference (HC)

	Centiles							Centiles					
	**50th**		2.5th		97.5th			**50th**		2.5th		97.5th	

**HC (mm)**	**weeks**	**days**	weeks	days	weeks	days	**HC (mm)**	**weeks**	**days**	weeks	days	weeks	days

70	**12**	**2**	11	2	13	3	140	**18**	**0**	17	0	19	0
72	**12**	**3**	11	3	13	4	142	**18**	**1**	17	1	19	1
74	**12**	**4**	11	4	13	5	144	**18**	**2**	17	2	19	2
76	**12**	**5**	11	5	13	6	146	**18**	**3**	17	3	19	3
78	**12**	**6**	11	6	14	0	148	**18**	**4**	17	4	19	4
80	**13**	**0**	12	0	14	1	150	**18**	**5**	17	5	19	5
82	**13**	**1**	12	1	14	2	152	**19**	**0**	17	6	20	0
84	**13**	**3**	12	2	14	3	154	**19**	**1**	18	1	20	1
86	**13**	**4**	12	3	14	4	156	**19**	**2**	18	2	20	2
88	**13**	**5**	12	4	14	5	158	**19**	**3**	18	3	20	3
90	**13**	**6**	12	5	14	6	160	**19**	**4**	18	4	20	4
92	**14**	**0**	12	6	15	0	162	**19**	**5**	18	5	20	5
94	**14**	**1**	13	1	15	1	164	**19**	**6**	18	6	20	6
96	**14**	**2**	13	2	15	3	166	**20**	**0**	19	0	21	0
98	**14**	**3**	13	3	15	4	168	**20**	**1**	19	1	21	1
100	**14**	**5**	13	4	15	5	170	**20**	**2**	19	2	21	2
102	**14**	**6**	13	5	15	6	172	**20**	**3**	19	3	21	3
104	**15**	**0**	13	6	16	0	174	**20**	**4**	19	4	21	4
106	**15**	**1**	14	1	16	1	176	**20**	**5**	19	5	21	5
108	**15**	**2**	14	2	16	2	178	**20**	**6**	19	6	21	6
110	**15**	**3**	14	3	16	4	180	**21**	**0**	20	0	22	0
112	**15**	**4**	14	4	16	5	182	**21**	**1**	20	1	22	1
114	**15**	**6**	14	5	16	6	184	**21**	**2**	20	2	22	2
116	**16**	**0**	15	0	17	0	186	**21**	**3**	20	3	22	3
118	**16**	**1**	15	1	17	1	188	**21**	**4**	20	4	22	4
120	**16**	**2**	15	2	17	2	190	**21**	**5**	20	5	22	4
122	**16**	**3**	15	3	17	3	192	**21**	**5**	20	6	22	5
124	**16**	**5**	15	4	17	5	194	**21**	**6**	20	6	22	6
126	**16**	**6**	15	5	17	6	196	**22**	**0**	21	0	23	0
128	**17**	**0**	16	0	18	0	198	**22**	**1**	21	1	23	1
130	**17**	**1**	16	1	18	1	200	**22**	**2**	21	2	23	2
132	**17**	**2**	16	2	18	2	202	**22**	**3**	21	3	23	2
134	**17**	**3**	16	3	18	3	204	**22**	**3**	21	3	23	3
136	**17**	**4**	16	4	18	5	206	**22**	**4**	21	4	23	4
138	17	6	16	6	18	6	208	**22**	**5**	21	5	23	5
							210	**22**	**5**	21	6	23	5

**Table 6:** Gestational age assessment by femur length (FL)

	Centiles					
	
	50th		2.5th		97.5th	
**FL (mm)**	weeks	days	Weeks	days	weeks	days

11	**11**	**5**	12	0	13	6
12	**12**	**1**	12	4	14	3
13	**12**	**4**	13	0	14	6
14	**13**	**0**	13	3	15	2
15	**13**	**3**	13	5	15	5
16	**13**	**5**	14	1	16	0
17	**14**	**1**	14	4	16	3
18	**14**	**4**	14	6	16	6
19	**14**	**6**	15	2	17	1
20	**15**	**2**	15	4	17	4
21	**15**	**4**	16	0	17	6
22	**15**	**6**	16	2	18	2
23	**16**	**2**	16	5	18	4
24	**16**	**4**	17	0	19	0
25	**16**	**6**	17	2	19	2
26	**17**	**1**	17	4	19	4
27	**17**	**4**	18	0	20	0
28	**17**	**6**	18	2	20	2
29	**18**	**1**	18	4	20	4
30	**18**	**3**	18	6	20	6
31	**18**	**5**	19	1	21	2
32	**19**	**0**	19	3	21	4
33	**19**	**2**	19	5	21	6
34	**19**	**4**	20	1	22	1
35	**19**	**6**	20	3	22	3
36	**20**	**2**	20	5	22	5
37	**20**	**4**	21	0	23	1
38	**20**	**6**	21	2	23	3
39	**21**	**1**	21	4	23	5
40	**21**	**3**	21	6	24	0

In supplementary analyzes, we compared z-scores of gestational age in the two ethnical groups. For both groups, mean z-scores for each anatomical parameter was non-different from zero (General Linear Models, SPSS). Nor did inclusion of ethnicity as an independent variable in the functions, which describe the estimated mean gestational age, reveal significant effects. Mean z-scores of gestational age as function of HC in Fulani and Kirdi women were -0.02 (95% CI: -0.22, 0.18), SD 0.98, and 0.02 (95% CI: -0.19, 0.22), SD 1.05, respectively. Correspondingly, mean z-scores of gestational age according to BPDoo in Fulani and Kirdi were 0.00 (95% CI: -0.19, 0.19), SD 0.92, and 0.00 (95% CI: -0.22, 0.23), SD 1.15, respectively. For BPDoo mean gestational age z-scores in Fulani and Kirdi women were -0.02 (95% CI: -0.21, 0.17), SD 0.94 and 0.02 (95% CI: -0.21, 0.25), SD 1.18, respectively. For FL mean gestational age z-scores in Fulani and Kirdi women were -0.12 (95% CI: -0.39, 0.15), SD 1.30 and 0.12 (95% CI: -0.08, 0.32), SD 0.97, respectively.

**Figure 5:**
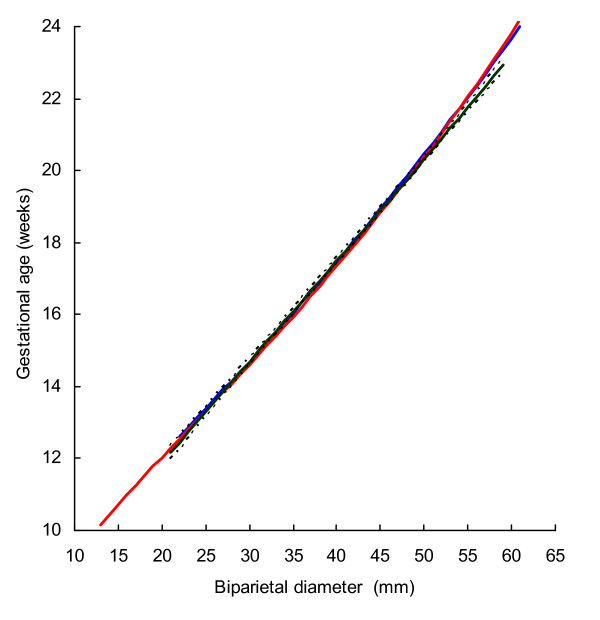
**The 50^th ^centile for biparietal diameter (outer-outer) with 95% confidence interval in the present study compared with those of Altman and Chitty (blue line)**[10]**and Johnsen *et al *(red line)**[14].

**Figure 6:**
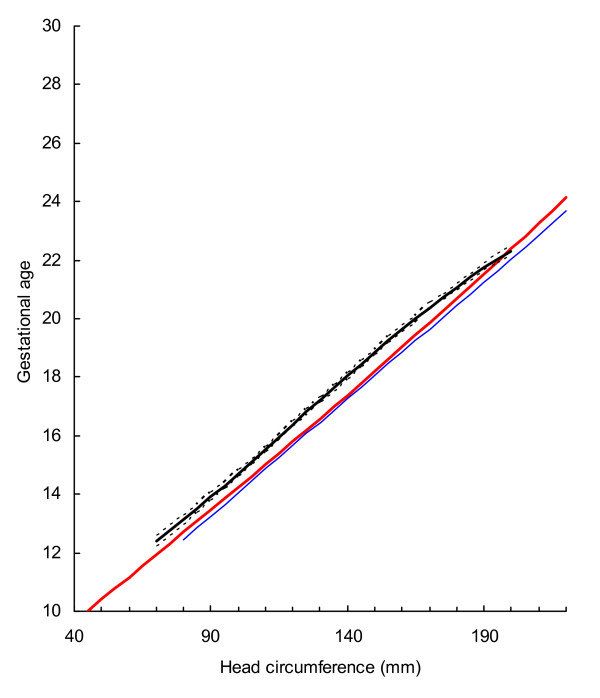
**The 50^th ^centile for head circumference with 95% confidence interval in the present study compared with those of Altman and Chitty (blue line)**[10]**and Johnsen *et al *(red line)**[14].

**Figure 7:**
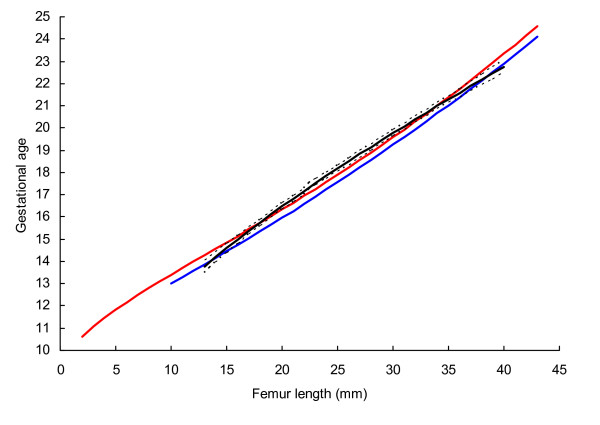
**The 50^th ^centile for femur length with 95% confidence interval in the present study compared with those of Altman and Chitty (blue line)**[10]**and Johnsen *et al *(red line)**[15].

Prediction of gestational age from BPD (22–59 mm) was fairly similar in three studies using outer-outer measurement technique (Fig 5). In practical terms, differences in predicted gestational age between the British and the Norwegian studies compared with the present study ranged from -0.03 to 0.4 weeks and -0,2 to 0.5 weeks, respectively. The 95% CI for the mean in the present study generally overlapped with the means of the other two studies. Predicted gestational age in the present study from HC (80–200 mm) was generally higher than those in the British and the Norwegian (Fig. 6). The difference from the British and the Norwegian studies ranged from -0.7 to -0.3 and -0.7 to 0.07 weeks, respectively. Predictions of gestational age from FL (13–40 mm) in the present and the Norwegian studies were similar and the difference ranged from -0.3 to 0.6 weeks (Fig. 7). However, predicted gestational age in the British study was generally lower than in the present (difference -0.6 to -0.1 weeks). The 95% CI for the mean in the present study generally overlapped with the mean in the Norwegian study. Design and statistical methods are comparable in these three studies. The 5^th ^and 95^th ^centiles were used to reflect uncertainty of gestational age estimation in the three methods (Table 7).

**Table 7:** Uncertainty of gestational age assessment, expressed as the distance between the 5^th ^and 95^th ^centile, when using outer-outer biparietal diameter (BPDoo), head circumference (HC) and femur length (FL) based on the present study, the study of Altman and Chitty [10] and that of Johnsen et al. [14]

		**Present study**		**Altman and Chitty**		**Johnsen *et al***	
	
	Measurement	50th centile	Uncertainty	50th centile	Uncertainty	50th centile	Uncertainty
	(mm)	(weeks + days)	(± days)	(weeks + days)	(± days)	(weeks + days)	(± days)
BPDoo	22	12 + 3	6	12 + 4	7	12 + 3	6
	50	20 + 2	5	20 + 3	13	20 + 2	9

HC	90	13 + 6	6	13 + 2	5	13 + 3	6
	180	21 + 0	6	20 + 5	10	20 + 4	8

FL	14	14 + 1	7	14 + 1	7	14 + 3	6
	32	20 + 2	8	20 + 0	11	20 + 2	8

In Tables 8, 9 and 10 the effects of maternal characteristics on fetal age assessment are presented. There was no significant effect of weight, height, maternal age or BMI on fetal biometry, but parity seemed to increase fetal BPDoo for the first three babies (p = 0.01), but not for HC or FL (p = 0.01, 0.27 and 0.11, respectively).

**Table 8:** Effects of maternal ethnicity, weight, height, body mass index (BMI), parity, and age on biparietal diameter outer – outer (BPDoo)

Maternal factor	Mean	95%CI		SD	p-value
Ethnicity					0.82
Fulani	40.52	40.19	40.85	9.8	
Kirdi	40.47	40.15	40.78	11.2	

Weight (centiles)					0.39
< 10	40.30	39.64	40.97	10.1	
10–90	40.44	40.18	40.69	10.6	
90+	41.11	40.40	41.83	10.6	

Height (centiles)					0.83
< 10	40.47	39.77	41.17	9.7	
10–90	40.54	40.27	40.80	10.7	
90+	40.25	39.64	40.86	10.6	

BMI (centiles)					0.33
< 10	40.47	39.74	41.19	8.5	
10–90	40.44	40.19	40.69	10.7	
90+	40.91	40.19	41.63	10.7	

Parity					0.01
0	38.67	37.97	39.38	11.4	
1	39.04	38.39	39.69	10.1	
2	39.70	38.85	40.55	11.2	
3	40.41	39.55	41.27	11.5	
4	39.45	38.51	40.40	10.3	
5+	39.99	39.12	40.87	9.3	

Age					0.10
-19	40.25	39.16	41.35	12.3	
20–24	39.98	39.37	40.58	10.4	
25–29	39.53	39.01	40.06	10.0	
30–34	38.55	37.81	39.28	11.9	
35+	39.41	38.28	40.55	9.7	

**Table 9:** Effects of maternal ethnicity, weight, height, body mass index (BMI), parity, and age on head circumference (HC)

Maternal factor	Mean	95%CI		SD	*p-value*
Ethnicity					0.89
Fulani	136.59	135.21	137.97	36.6	
Kirdi	136.46	135.13	137.78	40.4	

Weight (centiles)					0.22
< 10	135.47	132.66	138.27	39.4	
10–90	136.30	135.23	137.37	38.2	
90+	139.45	136.43	142.46	39.0	

Height (centiles)					0.58
< 10	136.10	133.17	139.03	37.5	
10–90	137.05	135.95	138.14	39.6	
90+	134.00	131.46	136.55	39.6	

BMI (centiles)					0.20
< 10	135.68	132.62	138.73	32.9	
10–90	136.42	135.35	137.49	38.8	
90+	138.19	135.16	141.22	38.1	

Parity					0.27
0	132.01	129.21	134.81	41.8	
1	131.25	128.68	133.82	36.3	
2	132.64	129.26	136.02	40.9	
3	135.78	132.38	139.17	40.8	
4	132.05	128.30	135.81	40.0	
5+	134.50	131.04	137.97	34.0	

Age					0.34
-19	134.81	130.45	139.16	45.6	
20–24	134.26	131.85	136.66	38.1	
25–29	132.09	130.00	134.17	36.7	
30–34	132.44	129.52	135.36	43.1	
35+	131.60	127.09	136.11	33.9	

Analysis of variance (ANOVA), adjustments for gestational age.

Parity and age were adjusted for each other.

**Table 10:** Effects of maternal ethnicity, weight, height, body mass index (BMI), parity, and age on fetal femur length (FL)

Maternal factor	Mean	95%CI		SD	*p-value*
Ethnicity					0.24
Fulani	24.53	22.89	26.17	7.7	
Kirdi	25.79	24.18	27.41	8.0	

Weight (centiles)					0.20
< 10	23.33	19.95	26.72	8.4	
10–90	25.26	23.96	26.57	7.8	
90+	26.53	22.97	30.08	7.8	

Height (centiles)					0.22
< 10	24.05	20.66	27.44	8.4	
10–90	25.18	23.84	26.52	7.8	
90+	26.04	22.99	29.09	7.6	

BMI (centiles)					0.60
< 10	21.61	17.99	25.24	7.3	
10–90	25.45	24.17	26.73	7.7	
90+	26.42	22.89	29.95	8.3	

Parity					0.11
0	23.95	23.02	24.88	8.7	
1	24.21	23.37	25.05	7.7	
2	24.77	23.62	25.92	7.7	
3	23.88	22.77	24.99	5.6	
4	24.63	23.40	25.86	8.2	
5+	25.63	24.53	26.73	7.4	

Age					0.17
-19	25.32	23.91	26.74	8.6	
20–24	24.91	24.15	25.66	7.8	
25–29	24.62	23.98	25.27	7.9	
30–34	23.99	23.03	24.96	8.3	
35+	23.71	22.27	25.15	7.1	

Analysis of variance (ANOVA), adjustments for gestational age.

Parity and age were adjusted for each other.

The intra-observer variation, calculated as the coefficient of variation for BPDoo, BPDoi, HC and FL was 2.7 (95% CI 2.5–2.9), 2.7 (2.6–2.9), 3.8 (2.8–3.3) and 3.1 (2.8–3.3), respectively. The corresponding intra-class correlation was 99.1% (95% CI 98.9–99.3), 99.2 (99.0–99.4), 98.4 (97.9–98.7) and 99.3 (99.11–99.4), respectively.

## Discussion

We have established reference charts for gestational age assessment using three fetal ultrasonographic measurements in an African population composed of two different ethnic groups. We have shown that significant morphometric ethnic differences had no significant influence on gestational age assessment. The charts are in agreement with European charts based on corresponding design and statistics.

Although ethnic groups (Kirdi and Fulani) differed significantly with respect to maternal age, height, weight and BMI (Table 1) we found no significant impact of ethnicity on fetal size at 12–22 weeks of gestation (Tables 8, 9 and 10). Additionally, supplementary analyses revealed no significant difference in distributions of z-scores of BPD, HC, or FL. Nor did we find any significant effects of ethnicity on predicted gestational age. These findings are very helpful in a country such as Cameroon, which has a variety of ethnic groups. It would be impractical to use different charts for all these groups. However, other studies [18-20] report clinically significant inter-ethnic morphometric differences, and a study among a multi-ethnic population in USA suggested accordingly that ethnicity and sex difference should be take into consideration to improve the accuracy of ultrasound estimation of GA [19]. However, we compared our new charts with those established for a Caucasian population and a mixed population in Europe [10,14,15] (Figures 5, 6 and 7) and found agreement for BPDoo-charts, the most commonly used measurement for this purpose. The method of assessing HC was different in Cameroon compared to the European studies (estimating circumference based on BPD and OFD compared to tracing or adjusting an ellipse to the fetal skull), which may explain some of the variance (Fig 6). The slightly different curvature of the means (Figures 6 and 7) may reflect that the fitted regression line would be different for the short time span of the present data compared the longer span of the other two studies.

As for FL there was no difference compared with the Norwegian study (95% CI overlapped). These two studies used identical insonation and measurement techniques as all ultrasound operators had been trained in the same unit, but then applied this technique using different machines in different countries. In general, FL charts vary more from study to study than the fetal head biometry, which probably reflects an uncertainty in defining the landmarks for the femoral diaphysis. We believe the small differences compared with the British charts are due to measurement technique, since study design and statistical methods were otherwise identical.

We have previously shown that morphometric differences at 18 weeks of gestation are related to body composition at birth [27]. Here we show that the ethnic impact on fetal morphometry at this stage of pregnancy is insignificant in the context of assessing gestational age. We acknowledge that ethnic differences are expressed during fetal development, but then mainly during the latter half of pregnancy. However, ethnical differences in mid gestation have been found for femur measurements in some studies [10,18-20], and we therefore recommend fetal age assessment in the second trimester to be based primarily on fetal head-measurements.

The impact of weight, height, maternal age, and BMI on fetal biometry, was non-existent in the present study or so small that it can be ignored before 22 weeks of gestation. There was a significant effect of parity on BPDoo, but since there was no effect on HC and FL it may well be due to chance. A study of normal pregnancies in Papua New Guinea found that fetal biometry was hardly affected by socio-demographic characteristics, weight gain during pregnancy or the height of the mother [3]; which is in line with the present findings.

In the present study all participants were married and lived with their husband, a sign of couple's stability. Although most of the women were housewives and dependent on their husbands, at least one member of the couple had income. This does not reflect the general population where more than 30% are believed to be unemployed, and this suggests that our study population was skewed. The fact that we included only women who knew their LMP probably augmented this skewed distribution as destitute pregnant women tend not to know their LMP and are therefore more likely to be excluded from the study. Recently established WHO standards for infant growth included children from optimal socioeconomic backgrounds at different locations around the world[28]. The present study should then be in line with such guidelines.

This study can be criticised for lacking perinatal data. We could not collect the outcomes of pregnancy as this would have necessitated the investigator's presence at the study site for a longer period. Home birth is common and would have resulted in numerous dropouts. Secondly, if growth deviation or other complications had occurred, we would not have excluded these participants [24]. Constructing reference charts by excluding participants for complications occurring after enrolment is not considered prudent, and carries the risk of constructing "supernormal" reference charts that are not applicable for women with complications.

## Conclusion

In countries such as Cameroon where the illiteracy is very high (68%) [1], only a few women know the date of their LMP. In general, even when educated, many women do not remember, or are uncertain of the date of their LMP [29]. Most pregnant women come to their first consultation around three months of gestation or later. GA assessed at this stage forms the base for growth assessment during the rest of the pregnancy and prediction of expected date of delivery. This study provides the tool for assessing GA by fetal biometry and makes it possible to determine IUGR and prematurity in Cameroon and other African populations. Although ultrasound machines are not readily available in antenatal care in developing countries, we believe that accurately assessed GA in risk groups would be important information at a time when ultrasound becomes increasingly available in these countries.

## Competing interests

The authors declare that they have no competing interests.

## Authors' contributions

DS contributed to conception and design of the study, acquisition of data, interpretation of data, revision of the manuscript, and approved the final version. TK contributed to conception and design of the study, analysis and interpretation of data, helped to draft the manuscript, and approved the final version. SR contributed to conception and design of the study, analysis and interpretation of data, helped to draft the manuscript, and approved the final version. SLJ contributed to analysis and interpretation of data, drafted the manuscript, and approved the final version.

## Pre-publication history

The pre-publication history for this paper can be accessed here:


